# Intermittent Fasting with or without Exercise Prevents Weight Gain and Improves Lipids in Diet-Induced Obese Mice

**DOI:** 10.3390/nu10030346

**Published:** 2018-03-12

**Authors:** Robin A. Wilson, William Deasy, Christos G. Stathis, Alan Hayes, Matthew B. Cooke

**Affiliations:** 1 College of Health and Biomedicine, Victoria University, Melbourne, VIC 3000, Australia; robin.wilson1@live.vu.edu.au (R.A.W.); william.deasy@live.vu.edu.au (W.D.); christos.stathis@vu.edu.au (C.G.S.); alan.hayes@vu.edu.au (A.H.); 2 Australian Institute for Musculoskeletal Science (AIMSS), Western Health, Melbourne, VIC 3021, Australia; 3 Department of Health and Medical Sciences, Swinburne University of Technology, Melbourne, VIC 3122, Australia

**Keywords:** high fat diet, weight loss, energy balance, body composition, fasting glucose, plasma lipids

## Abstract

Intermittent fasting (IF) and high intensity interval training (HIIT) are effective lifestyle interventions for improving body composition and overall health. However, the long-term effects of IF and potential synergistic effects of combining IF with exercise are unclear. The purpose of the study was to investigate the long-term effects of IF, with or without HIIT, on body composition and markers of metabolic health in diet-induced obese mice. In a randosmised, controlled design, 8-week-old C57BL/6 mice (males (*n* = 39) and females (*n* = 49)) were fed a high fat (HF) and sugar (S) water diet (30% (*w*/*v*)) for 24-weeks but were separated into five groups at 12-weeks: (1) ‘obese’ baseline control (OBC); (2) no intervention (CON); (3) intermittent fasting (IF); (4) high intensity intermittent exercise (HIIT) and (5) combination of dietary and exercise intervention (IF + HIIT). Body composition, strength and blood variables were measured at 0, 10 and/or 12-weeks. Intermittent fasting with or without HIIT resulted in significantly less weight gain, fat mass accumulation and reduced serum low density lipoproteins (LDL) levels compared to HIIT and CON male mice (*p* < 0.05). The results suggest that IF, with or without HIIT, can be an effective strategy for weight gain prevention despite concurrently consuming a high fat and sugar diet.

## 1. Introduction

Obesity is a complex multifaceted disease resulting from the interplay between genetics and lifestyle, including economic growth, modernization, and urbanization [1]. The rapid rise in obesity prevalence appears to be a reflection of the changes in dietary and behavioural patterns, with eating habits shifting to greater consumption of energy-dense foods that are high in fats and sugars, while at the same time, levels of physical activity are decreasing. These differences in energy intake and expenditure, often referred to as energy balance, have direct implications for weight regulation, with even small deviations in daily energy balance resulting in large body weight changes over the long term [2]. Interventions that target and reverse these small deviations in energy balance may be an effective tool in reducing body weight and adiposity, but also help maintain a stable body weight over the longer period.

Diet and exercise interventions aiming to shift the energy balance towards negative by either decreasing caloric intake and/or increasing physical activity have shown to be effective for weight loss [3]. Many iterations of such dietary and physical activity interventions have been proposed, but intermittent fasting (IF) and high intensity interval training (HIIT) have recently been purported as effective strategies [4,5]. Intermittent fasting (IF) is a dietary regime that refers to short periods of intense energy restriction (75–100% reduced caloric intake on fasting days) followed by ‘normal’ eating on non-fasting days [6,7]. Short-term intervention studies have shown that intermittent fasting can reduce body weight and fat mass, fasting glucose and insulin levels, and improve insulin sensitivity and lipid profiles [6,8]. Despite limited comparison studies, IF appears to present an alternative and equivalent option to the traditional diets involving continuous energy restriction as a way to improve body composition and other health markers [9].

High intensity interval training is promoted as a superior and time efficient method for reducing body and fat mass and other biomarkers of chronic diseases compared to moderate intensity continuous training. HIIT refers to brief bouts of vigorous intensities (80–90% VO2 max) interspersed by relatively longer bouts of low intensity active recovery or passive recovery [10,11]. The effectiveness of HIIT is its ability to produce rapid physiological adaptations that may promote greater energy deficit post-exercise, independent of the changes in total physical activity energy expenditure. Our lab previously reported higher plasma hypoxanthine accumulation and urinary purine base excretion (indirect markers of energy loss from the muscle) following high intensity exercise compared to continuous exercise [12]. These losses could indicate the need for greater energy input for subsequent restorative processes (via intramuscular purine de novo replacement) during the recovery period, and may in part contribute to the negative energy balance that leads to fat loss following long-term high intensity training programs [13,14]. Our findings and others [15,16] have suggested that training programs that include high intensity and low volume exercise could be effective at creating metabolic disturbances that can result in enhanced weight loss in the form of reduced adipose tissue, but also promote health and fitness benefits normally seen over the longer term.

Manipulating the energy balance equation through exercise and diet are effective strategies to improve body composition and manage lifestyle-related metabolic diseases. However, protocols that are characterised by severe energy restriction or large energy expenditure such as IF and HITT, respectively, may induce greater physiological changes and/or further stimulate already maximal adaptation rates, independent of changes in energy balance. The current study was designed to investigate the effect of IF and HIIT, alone and in combination, on anthropometric and metabolic health parameters in a model of diet-induced obese mice. We hypothesised that the combined interventions will result greater prevention of weight gain in the form of fat mass, reductions in fasting glucose levels and markers of insulin sensitivity and glucose tolerance, and improvement in lipid profile compared to either dietary and exercise intervention alone.

## 2. Materials and Methods

### 2.1. Animals

One hundred, 8-week old C57BL/6 mice (Avg. Wt: 22.2 ± 2.5 g Males, 17.6 ± 1.7 g Females) were acquired from the Animal Resource Centre (Murdoch, WA, Australia) and housed at the animal holding facility at Werribee campus, Victoria University. Mice were housed in groups of 5 at a constant temperature (22 °C), under a 12:12-h light-dark photoperiod with ad libitum access to food and water (other than fasting periods) in accordance with ethical approval obtained from the Victoria University Animal Ethics Committee, conforming to the Australian code of practice for the care and use of animal for scientific purposes. All animals were fed a high fat (HF) (59% energy from fat, SF03-002, Speciality Feeds, Glen Forrest, WA, Australia) and high sugar (S) water (30% sugar *w*/*v*) diet for a period of 24-weeks. The initial 12-weeks of HF/S feeding was designed to induce obesity within the mice according to protocol used by La Fleur [17].

**Figure 1:**
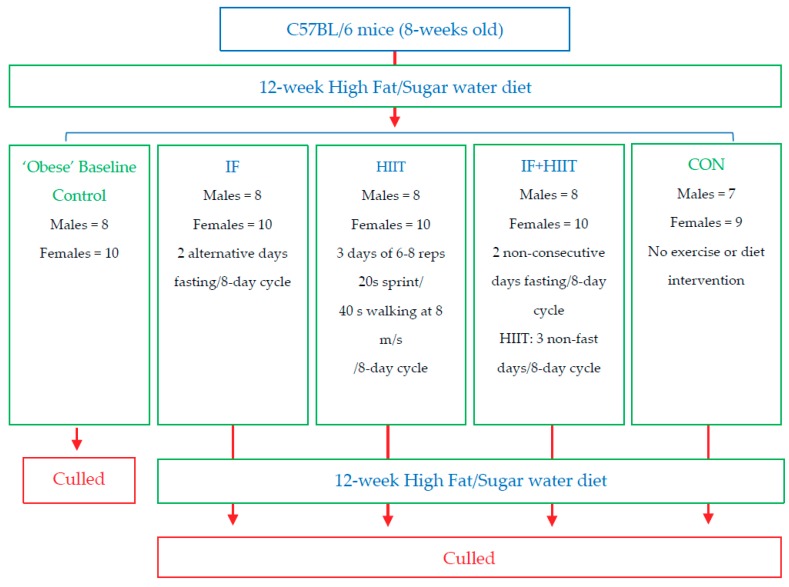
Flow diagram of study design.

One group of mice was killed after 12-weeks of HF/S feeding (Males = 8, Females = 10) to establish baseline measures for some variables to compare to the remaining groups. The remaining mice were randomly allocated using block randomisation (both allocator and executer blinded to the blocks) into 4 groups: no intervention (CON; Males = 7, Females = 9); intermittent fasting (IF; Males = 8, Females = 10), high intensity interval training (HIIT; Males = 8, Females = 10) and a combination of the dietary and exercise intervention (IF + HIIT; Males = 8, Females = 10) (Figure 1). These groups continued the HF/S feeding for another 12-weeks and after a total of 24-weeks of HF/S feeding and intervention, mice were sacrificed and tested as described below. To minimise any acute transient effects from either fasting and exercise treatment [18], a period of 48 h was given between the last fasting period and exercise session and any physiological measurement or sample collection for all animals. Eleven male mice died during the study intervention period. Of the 11 that died, 6 died during the HF/S dietary intervention period and before group allocation; 2 died from the IF group; 2 died from the HITT group; and 1 died from the combined dietary and exercise group. All mice underwent necropsy by the animal house technical staff with no obvious cause of death. Furthermore, these deaths were also reported to the animal ethics committee of University and cleared of any negligence on the researcher’s behalf and the experimental design of the study. One female mouse did not gain weight in the control group and was excluded from the analysis. All groups had ad libitum access to the HF/S diet. All intervention groups underwent an 8-day cycle. This was designed to give at least one-day break between fasting days and exercise days for combination (IF + HIIT) group and ensure animal wellbeing. To ensure uniformity between the groups, IF and HIIT groups underwent the same 8-day cycle. However, the total period of interventions was restricted to 12 calendar weeks.

### 2.2. Intermittent Fasting Protocol

Mice from IF and IF + HIIT group were deprived of food on two alternate days of an eight-day cycle for 12-weeks. During the fasting period, mice were moved to clean cages with potable water available ad libitum and returned to their original cages 24 h later. On non-fasting days, mice had ad libitum access to the HF/S diet [19,20]. Mice were always fasted on day 2 and 7 of the 8-day cycle.

### 2.3. Top Speed Test and High Intensity Interval Training Protocol

All groups were acclimatized to treadmill running through five gradual treadmill training familiarization sessions over a period of 2-weeks prior to intervention commencement according to standard operating procedures for treadmill exercise and university animal ethics guidelines. The protocol for top speed determination was as follows: mice performed an incremental warm up period for 5 min reaching 8 cm/s. The speed was slowly increased from 8 to 35 cm/s and was held at 35 cm/s for 20 s, and then returned back to 8 cm/s for 40 s. Following this, the speed was slowly increased to 40 cm/s and maintained for 20 s followed by another 40 s of active rest at 8 cm/s. The 20 s running, and 40 s active rest protocol was repeated with increasing running speed by 5 cm/s until the mouse was unable to continue running. The running speed prior to this was recorded as the top speed. The average top speed for all mice was 59 ± 2 cm/s. To minimise the effects of the training adaptation during the familiarisation sessions, all mice, irrespective of their group, underwent the 2-weeks familiarisation training.

The HIIT protocol chosen was a slightly modified protocol used by Tuazon, et al. [21] with the relatively low volume and high intensity shown to have positive effects on body composition and metabolic health [22]. Briefly, the HIIT training sessions consisted of a 5 min warm up period at 8 cm/s, followed by 6–8, 20 s bouts of treadmill running at the previously determined top speed of the individual animal, interspersed by 40 s active rest at 8 cm/s. The slope of treadmill was maintained at 0°. To implement overload, the number of intervals was increased from 6 at the beginning of the training intervention to 8 at the end of the eighth HIIT session. HIIT was performed on three non-consecutive days per 8-day cycle. To encourage the mice to run on the treadmill at their maximum speed, an air puff device was used if the mice appeared to slow down.

### 2.4. Body Weight and Composition Assessment

Body weight was measured weekly on laboratory weighing balance. Body composition (adipose and lean mass) was determined using EchoMRI (EchoMRI- body composition analyser, Houston, TX, USA) before and after 10-weeks of diet and/or exercise intervention.

### 2.5. Assessment of Fasting Blood Glucose and Glucose Tolerance (AUC)

Fasting blood glucose and glucose tolerance was analysed before and after 10-weeks of diet and/or exercise intervention. Fasting glucose was measured using handheld glucometer (Accu-Check GO glucometer, Roche Diabetes Care, Inc., Indianapolis, IN, USA) from blood collected by snipping the tail tip (1–2 mm) following an overnight (12 h) fast and between the hours of 7 a.m.–9 a.m. Mice were given intraperitoneal injection of glucose (1.5 g/kg body weight) and blood was collected from the tail before and 5, 15, 30, 45, 60, 90 and 120 min following the glucose administration. Glucose tolerance was calculated as glucose area under the curve (AUC) using trapezoidal method [23].

### 2.6. Assessment of Plasma Insulin, Glucose and Triacylglycerol (TAG), Low-Density Lipoprotein (LDL) and High-Density Lipoprotein (HDL) Levels

Plasma insulin, glucose, TAG, LDL and HDL were measured after 12-weeks of diet and/or exercise intervention. Following 12 h fasting, mice were anaesthetised with intraperitoneal injection of pentobarbitone (60 mg/kg). A total of 48 h was given between the final exercise session or IF period and culling day. Blood was collected by cardiac puncture and plasma was prepared using EDTA as anticoagulant and centrifugation at 2000× *g* for 15 min. Plasma glucose was determined by handheld glucometer (Abbott Freestyle Optium, Abbott Diabetes Care, Ltd., Oxfordshire, UK). Plasma insulin, LDL and HDL was analysed using commercially available kits (Crystal Chem, Inc., Downers Grove, IL, USA). Plasma TAG was analysed using commercially available kits (ABCAM, Cambridge, MA, USA).

### 2.7. Assessment of Homeostatic Model Assessment of Insulin Resistance (HOMA-IR)

The homeostatic model assessment of insulin resistance (HOMA-IR) was determined by using the following formula: fasting plasma insulin (mU/L) × fasting plasma glucose (mmol/L)/22.5 [9].

### 2.8. Assessment of Muscle Strength

Muscle strength was measured before and after 10-weeks of diet and/or exercise intervention using a grip strength test as previously described by Deacon [24]. Briefly, a grip test apparatus was used which consisted of a of stainless steel wire ball connected to a series of steel chain links, varying from one (15 g) to six (74 g). Mice were held by the base of their tail and allowed to grasp the grip test apparatus lying on a lab bench with its forepaws. Mice were lifted until the apparatus was raised from the bench. Holding the apparatus at a specific weight for 3 s was deemed a successful lift and the mice would proceed to next heavier weight. If the mice failed to achieve the 3 s target for 5 consecutive times, the trial was stopped, and mouse was assigned the weight/time achieved. A 30 s rest between lifts was used. The final score (absolute muscle strength) was calculated by the number of links (1 to 6) in the last heaviest weight held for 3 s multiplied by time i.e., 3, plus the number of seconds last heaviest weight was held for less than 3 s. The total score was divided by the respective lean mass to calculate relative muscle strength. This method was adopted to measure the changes in muscle strength rather than the inverted screen test often used for testing grip strength, which is affected by total body weight.

### 2.9. Assessment of Caloric Intake

Food and sugar water intake of animals from each cage was measured weekly and average caloric intake per animal per day was calculated. Due to housing constrains of ethical approval and to avoid added stress, animals from the control and HIIT groups were housed together and animals from IF and IF+HIIT groups were housed together. The average caloric intake from the intervention control + HIIT animal (“non-fasting” group) was compared to IF + IF + HIIT animals (“fasting” group) to determine if intermittent fasting resulted in over compensation of food intake on non-fasting days over the 12-weeks intervention period.

### 2.10. Statistical Analysis

Data is presented as mean ± standard deviation (SD). Delta changes (pre- and post-intervention) in body weight and body composition (fat mass and lean mass), fasting blood glucose, glucose tolerance and muscle strength were analysed using 4 (CON, IF, HIIT, IF + HIIT) × 2 (0 and 10-weeks) repeated measures ANOVA. Tukey’s test was used to analyse any significant interaction effects. Since baseline measurements of plasma insulin, glucose and LDL, HDL and TAG levels were not taken for each intervention group, analysis of delta changes for plasma insulin, HOMA-IR and lipid panels were performed using the mean average of group 1 (HF/S ‘obese’ control values) as the “baseline” minus the post-intervention values of the respective intervention groups. These delta values were analysed using 4 (CON, IF, HIIT, IF + HIIT) × 2 (0 and 12-weeks) repeated measures ANOVA. One way ANOVA was also performed on plasma insulin, HOMA-IR and lipid panels at 12-weeks. Baseline variables and average caloric intake between groups was analysed using an independent students’ *t*-test. Sample size was calculated using G Power software. Based on the difference in weight gain between control HFD and alternative day fasting + HFD groups [25], the calculated effect size was 7.9. With alpha of 0.05, a sample size of 8 per group would yield a power of 0.95. A 5–10% loss was incorporated to give group size of 9. An alpha level of 0.05 was adopted throughout to reduce Type I statistical errors.

## 3. Results

### 3.1. Effects of 12-Weeks of the HF/S Diet on Body Weight of All Animals

A significant increase in body weight was observed in male and female mice following the initial 12-weeks of consumption of the HF/S diet (Male = Baseline: 22.2 ± 2.5 g, 12-week: 36.2 ± 2.1 g (~63%↑); Female = Baseline: 17.6 ± 1.7 g, 12-week: 28.5 ± 1.5 g (~62%↑)).

### 3.2. Effects of IF on Caloric Intake

No significant differences were observed in average caloric intake during the intervention period between the CON and HIIT (Non-Fasting) group and IF and IF + HIIT (Fasting) group in both male (14.63 ± 1.47 kcal/mouse/day versus 13.23 ± 1.62 kcal/mouse/day, *p* < 0.05) and female mice (11.93 ± 1.42 kcal/mouse/day versus 11.43 ± 1.58 kcal/mouse/day, *p* < 0.05).

### 3.3. Effects of IF and/or HIIT on Body Weight and Composition

**Figure 2:**
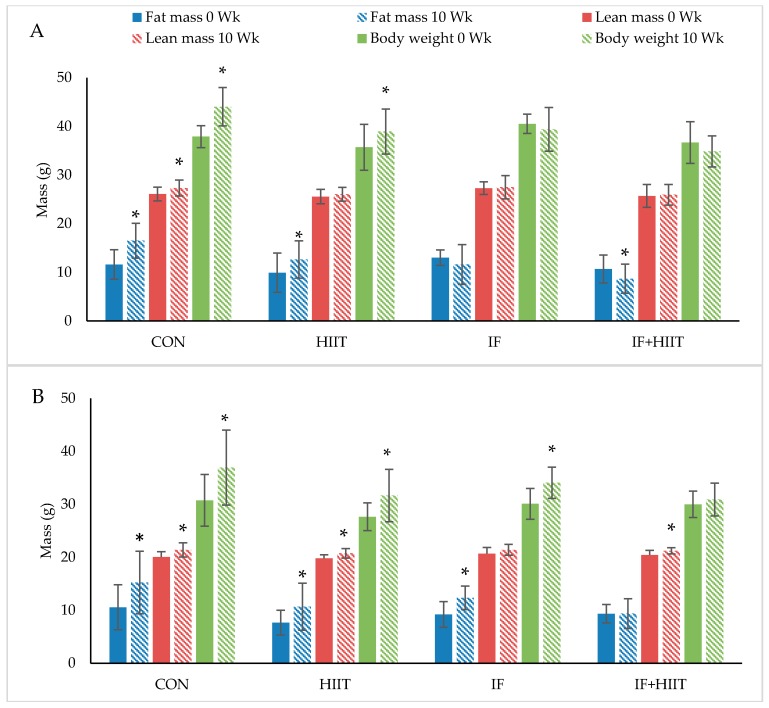
The effect of 10-weeks of intermittent fasting and high intensity exercise combined or individual on body weight (g), fat mass (g) and lean mass (g) in both male (**A**) and female mice (**B**). Bars represent means ± SD. * represents significant change over time.

**Figure 3:**
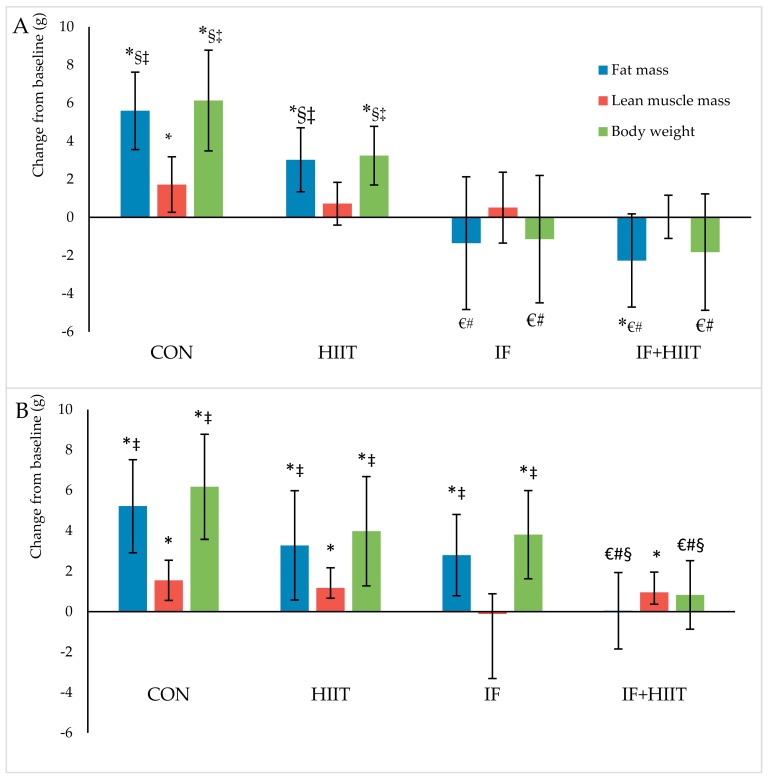
Body composition changes (g) from baseline in males (**A**) and females (**B**) after 10-weeks of diet and/or exercise intervention. Bars represent means ± SD. * significant change over time; € significantly different from CON; # significantly different from HIIT; § significantly different from IF; ‡ significantly different from IF + HIIT.

Body weight and composition measured before and after 10-weeks of diet and/or exercise intervention is presented in Figure 2A,B (Absolute values) and Figure 3A,B (change from baseline).

#### 3.3.1. Body Weight and Fat Mass

Following the initial 12-weeks of HF/S diet, body weight and fat mass continued to significantly increase over time in the CON and HIIT groups for both male and female mice (*p* < 0.05), and IF female group only (*p* < 0.05). After 10-weeks of the diet and/or exercise intervention, male mice displayed significantly lower body weights and reduced fat mass in the IF and IF + HIIT groups compared to the CON (*p* < 0.05) and HIIT groups (*p* < 0.05) (Figure 2A). In the female mice, the IF + HIIT group displayed significantly lower body weights and reduced fat mass compared to CON (*p* < 0.05), IF (*p* < 0.05) and HIIT groups (*p* < 0.05) (Figure 2B).

#### 3.3.2. Lean Mass

Lean mass significantly increased over the 10-weeks diet and/or exercise intervention period in the CON group for both male and female mice (*p* < 0.05) and in the HIIT and IF + HIIT female groups only (*p* < 0.05) (Figure 2A,B). No significant differences were observed between groups at the end of the diet and/or exercise intervention period.

### 3.4. Effect of IF and/or HIIT on Blood Glucose, Plasma Insulin, HOMA-IR and AUC

**Figure 4:**
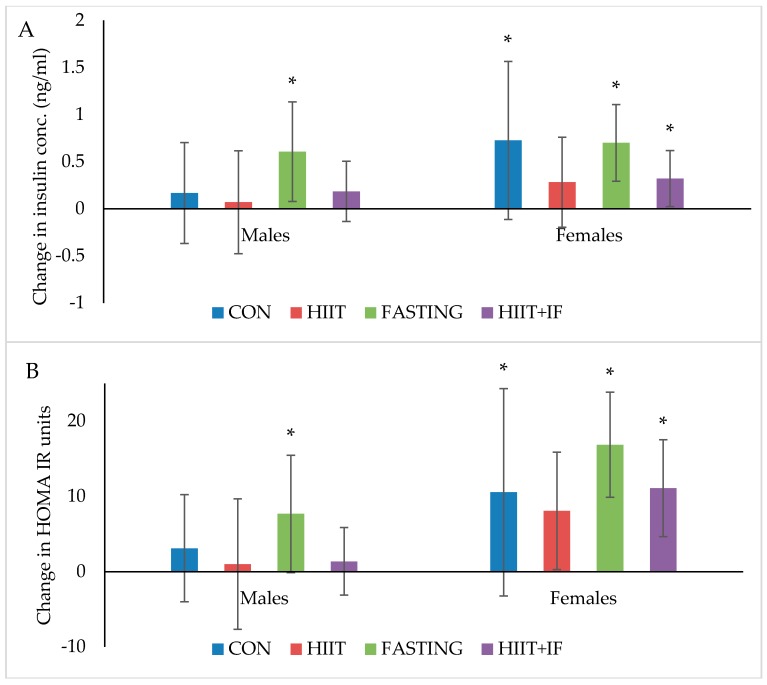
(**A**) Insulin (ng/mL) and (**B**) HOMA-IR changes from ‘obese’ baseline in males and females after 12-weeks of diet and/or exercise intervention. Bars represent means ± SD * significant change over time.

Blood glucose and AUC measured before and after 10-weeks of diet and/or exercise intervention is presented in Table 1. Changes in plasma insulin and HOMA-IR levels following 12-weeks of diet and/or exercise intervention is presented in Figure 4A,B, respectively.

#### 3.4.1. Blood Glucose

Fasting blood glucose levels significantly increased in the CON and IF male groups over the 10-week diet and/or exercise intervention period (*p* < 0.05, Table 1). At the end of the 10-week diet and/or exercise intervention period, the IF group for both male and female mice displayed significantly higher fasting glucose levels compared to the other three groups (*p* < 0.05).

#### 3.4.2. Plasma Insulin

Plasma insulin levels significantly increased over the 12-week diet and/or exercise intervention period in both male and female IF groups (*p* < 0.05) and female CON (*p* < 0.05) and IF + HIIT (*p* < 0.01) groups only (Figure 4A). No significant differences between groups were observed at the end of the diet and/or exercise intervention period.

#### 3.4.3. Insulin Resistance (HOMA-IR) and Glucose Tolerance (AUC)

HOMA-IR, a marker of insulin resistance significantly increased over the 12-week diet and/or exercise intervention period in the male and female IF groups (*p* < 0.05, Figure 4B). However, similar to changes in plasma insulin, female mice also showed a significant increase in HOMA-IR in the CON (*p* < 0.05) and IF + HIIT groups (*p* < 0.05). In addition, female mice demonstrated a significant increase in AUC (Table 1), a marker of glucose tolerance in the IF and IF + HIIT groups over a 10-week diet and/or exercise interventions period (*p* < 0.05, Table 1). No significant differences between different groups were observed at the end of the diet and/or exercise intervention period.

### 3.5. Effect of IF and/or HIIT on Lipid Profiles

Plasma LDL, HDL and TAG levels measured at 0-week or at the end of the 12-week diet and/or exercise intervention period is presented in Table 2.

In female mice, plasma LDL levels significantly decreased over the 12-week diet and/or exercise intervention period in all groups (*p* < 0.05). Conversely, HDL significantly increased over 12 period, but only in the HIIT and IF groups (*p* < 0.05). In male mice, LDL levels significantly decreased over the 12-week diet and/or exercise intervention period in the IF and IF + HIIT groups (*p* < 0.05), whereas CON and HIIT groups significantly increased over that period (*p* < 0.05). At the end of the intervention period, plasma LDL levels were significantly lower in the male IF and IF + HIIT groups compared to CON (*p* < 0.05) and HIIT (*p* < 0.05) groups. No significant differences were observed for plasma HDL and TAG levels (Table 2). In the female mice, plasma TAG levels were significantly lower in the IF+HIIT group compared to HIIT group at the end of the 12-week period (*p* < 0.05). No significant differences between different groups were observed for plasma LDL and HDL levels in females (Table 2).

### 3.6. Effect of IF and/or HIIT on Muscle Strength

Absolute and relative muscle strength measured before and after 10-weeks of diet and/or exercise intervention is presented in Table 3. Absolute muscle strength was significantly increased in the CON group over the 10-week period in the male mice (*p* < 0.05). In the female mice, absolute muscle strength was decreased in the IF group over the 10-week period (*p* < 0.05). Similarly, when muscle strength was expressed per lean mass, relative muscle strength also declined over the 10-week period in IF females group only. No other significant differences were observed for absolute muscle strength or relative muscle strength (Table 3).

## 4. Discussion

Intermittent fasting with or without high intensity interval training resulted in significantly less weight gain in male mice despite concurrently consuming a high fat and sugar diet. The reduced weight gain was predominantly in the form of lower fat mass accumulation, with no loss in lean mass. At the end of the diet and/or exercise intervention period, LDL levels were significantly lower in the male IF and IF + HIIT groups compared to controls, whereas female mice demonstrated significantly lower TAG levels in the IF + HIIT group compared to HIIT group. Interestingly, intermittent fasting appeared to have a negative impact on markers of glycaemic control, especially within the female mice, with significant elevations in fasting blood glucose, glucose tolerance and HOMA-IR observed at the end of the intervention period. The changes in body composition, glycaemic control and lipid panels seem to be gender specific with male mice demonstrating greater changes in most variables compared to the female mice, independent of the intervention.

Following an initial 12-weeks of HF/S feeding to induce obesity, a further 10-weeks of HF/S diet caused a significant increase in body mass and fat mass by ~16% and ~47%, respectively in CON male mice and by ~19% and ~46%, respectively in CON female mice. These changes are comparable to those reported elsewhere using a similar diet [26,27]. Combining IF for 2 days per week with HIIT for 3 days per week produced the most effective changes in body weight and fat mass compared to either IF or HIIT intervention alone. The IF + HIIT group weighed ~9 g and ~6 g less and displayed ~66% and 24% lower fat mass in the males and females, respectively, compared to the CON mice. The reduced weight gain and fat accumulation was also greater compared to the HIIT and IF groups; although not significantly different between the male IF groups. The body compositional changes observed in the combined diet and exercise group supports the primary hypothesis of the study and provides evidence of an additive affect when implemented concurrently. Studies using combined CR with exercise have also reported reduced weight gain in comparison to control animals (no diet and exercise) [28,29]. Huffman and colleagues [29] administered 24-weeks of CR (18% restriction of high fat diet) combined with five days per week of treadmill running at speeds approximately 13–15 m/min resulted in weight gain prevention (~15 g) and fat mass reductions (~45%) compared to CR only. Similar weight gain prevention (~15 g) was also observed following 30% CR combined with voluntary wheel running (distance not measured) for 8-weeks [28]. These changes, especially in weight, were greater than the body compositional changes observed in the current study and are likely due to a larger energy deficiency created by the greater energy restriction and/or higher exercise-induced energy expenditure of the protocols used [28,29].

The intermittent fasting intervention also resulted in significantly less weight gain (~18% or ~7.3 g) and fat mass accumulation (~58% or ~6.9 g) compared to CON group; albeit only in male mice. Previous studies using ADF regimes have also shown improvements in body composition, with reduced gains in body mass over a 4- to 6-week period compared to mice consuming a standard chow diet [22,30]. Using a similar model of diet-induced obesity to that of the current study, 4-weeks of ADF led to lower body mass and fat mass gains by approximately 13% and 50%, respectively, compared to high fat fed controls [26]. Similarly, Higashida and colleagues [25] also showed significantly less weight gain (~27%) and intra-abdominal fat accumulation (~39%) following 6-weeks of ADF compared to the high fat fed controls in rats [25]. The greater difference in reduced weight gain reported in the Higashida et al. [25] study versus the present study (~27% vs. ~16%) could be due to the frequency of fasting days, as the Higashida et al. [25] implemented their 21 days of fasting within a 6-week period, whereas the current study implemented 24 days of fasting within a 10-week period. Indeed, Joslin and colleagues [18] demonstrated greater changes in body composition, in fact, weight loss, following 35 fasting days within a 10-week ADF protocol. Notwithstanding, it is evident from the current study and others that IF is an effective method of reducing body weight and fat mass in mice consuming either standard chow or a high fat and sugar diet. The results are most likely a reflection of the substantial physiological and biochemical changes created by the negative energy balance following fasting periods.

It should be highlighted that despite the intermittent fasting group with or without exercise demonstrating reduced weight gain compared to HF/S diet group only, only modest changes in weight occurred within each group. This could be explained by the recent findings of the MATADOR study which demonstrated greater weight loss efficiency when intermittent energy restriction was interrupted with periods of energy balance ‘rest periods’ to potentially reduce any compensatory metabolic responses that can occur to return the body to its original weight [31]. Whether such compensatory mechanisms explain the modest weight changes observed in the current study requires further investigation.

Another strategy to create a negative energy balance is to increase energy expenditure greater than energy intake [32]. In the current study, mice completing 10-weeks of HIIT, 3 times a week displayed less body mass and fat mass by approximately 5% and 8% and 6% and 10% in male and female mice, respectively compared to CON mice. Greater changes in weight and fat mass have been observed in other studies with up to 16% and 45% prevention in weight and fat gain, respectively [22,33]. However, such differences are most likely due to the training volume, with other studies undertaking 10–12 bouts of HIIT, five days a week for 8 to 10-weeks [22,33] compared to the present study of eight bouts, three days per week. Furthermore, the HIIT group in the present study concurrently consumed a diet high in fat and sugar during the intervention period and thus, any change in appetite (i.e., increase) because of the exercise training would have resulted in increased consumption. Consuming a standard chow diet may have attenuated weight gain or perhaps led to weight loss.

Glucose intolerance, insulin resistance and elevated TAG and LDL levels have been noted in animal models of diet-induced obesity [22,26,34]. In the current study, high fat feeding resulted in hyperglycaemia and hyperlipidaemia, insulin resistance and glucose intolerance in both male and female ‘Obese’ baseline control mice, with elevated glucose levels indicating diabetes onset. Despite previous studies demonstrating improved glycaemic control and reduced diabetes risk following even modest weight reduction [26,33], the present study was unable to confirm such findings following the diet and/or exercise interventions. Lower LDL and TAG levels following the combined interventions in both males and female mice, respectively, compared to controls was observed at the end of the intervention period. The effect of IF on glucose metabolism and glycaemic control was unexpected. Both male and female IF groups exhibited significant increases in fasting glucose and plasma insulin and glucose tolerance and insulin resistance at either 10- or 12-weeks. Although these results are contradictory to the commonly reported view that IF lowers fasting blood glucose levels and improves glycaemic control [6], it has been reported that long term (32-weeks) of IF, but not CR, can lead to redox imbalances, insulin receptor nitration, and thus glucose intolerance in rats [35]. Reduced glucose tolerance is usually a result of lower insulin release [36], however, in the current study, insulin levels were significantly higher than the ‘obese’ controls at the end of the intervention period. It is difficult to make comparisons to other animal and human IF studies since the mice in the current study concurrently consumed a HF/S diet, rather than a typical standard chow or ‘healthy’ diet often prescribed in animal and human studies, respectively [37,38]. A study by Joslin et al., [18] did report improved glucose tolerance and lower circulating insulin levels following ADF with concurrent HF feeding. The improvements observed in the Joslin et al. study could be due to the greater weight loss reported (~16 g) compared to the current study (1.14 g), with evidence suggesting the amount of weight loss may drive the benefits in glucose handling in obesity [39].

Finally, the attrition rate reported in the current study was unforeseen. All mice underwent necropsy by the animal house technical staff with no obvious cause of death. This strain of mice (C57BL6) are a well-established model for investigating diet-induced obesity and type II diabetes. There is some evidence to suggest that male mice are more prone to the adverse effects of HFD, and that dietary and so-far-unidentified environmental factors can combine to induce obesity and diabetes of variable severity [40]. Whether this susceptibility contributed to the death of some of the male mice is unknown. Furthermore, given the quality of reporting in animal research, especially in regards to attrition rates, has been quite poor [41], it is difficult to compare our attrition rate to others of similar design.

## 5. Conclusions

Current evidence suggests that as little as 10 min of high intensity exercise can improve metabolic health and aerobic capacity [42] and alternative day fasting can reduce obesity-associated changes in body composition, fasting insulin and glucose concentrations [43,44]. The current study wanted to confirm the aforementioned findings by mimicking similar protocols in animal models. Secondly, to investigate the combined effects of such regimes and its long-term impact, and thirdly, unlike human studies which typically don’t change the research participant’s diet, we wanted to observe the effects of these diet and exercise regimes while concurrently consuming a high fat and sugar diet. The present study is the first study to demonstrate superior effects on body composition and lipid profiles following combined IF and HIIT compared to either diet or exercise intervention alone while concurrently consuming a high fat and sugar diet. These observations are likely due to the physiological and biochemical changes that occur when creating a negative energy balance shift. An observation of interest was the gender specific responses to the same diet and/or exercise intervention and could indicate potential hormonal differences influencing metabolic control/adaptation in mice. The results of the present study suggest that the combination of diet and exercise are most effective at attenuating the negative impact that high fat and sugar diet has on body weight, composition and lipid levels.

## 6. Limitations

A number of limitations exist in the current study. Firstly, due to limitations in obtaining adequate blood from the tail of the mice, only blood glucose and AUC were measured before and after 10-weeks of diet and/or exercise intervention. Plasma insulin, glucose, HOMA-IR and lipid panels were only measured at the end of the 12-week intervention period and subsequently compared to the average ‘obese’ baseline controls rather than the true baseline (0-week) for each individual intervention group to analyse changes over time. Given starting weights for all intervention groups and the ‘obese’ baseline group were not significantly different, we can only speculate that the hormonal and lipid levels would be similar. Secondly, due to housing constrains of ethical approval, mice from different intervention groups were housed together, more specifically, mice undergoing fasting (IF and IF + HIIT) were housed together and mice undergoing no fasting (HIIT and CON) were housed together. Thus, food intake of individual mice could not be attained.

## Figures and Tables

**Table 1:** The effect of 10-weeks of intermittent fasting and high intensity exercise combined or individual on fasting blood glucose and glucose tolerance (AUC) in both male and female mice.

Variables	Gender	CON		HIIT		IF		IF + HIIT	
		0 Week	10 Week	0 Week	10 Week	0 Week	10 Week	0 Week	10 Week
Fasting blood glucose (mmol/L)	Males	7.2 ± 0.9	8.8 ± 1.7 *	7.3 ± 1.4	7.8 ± 1.1	7.6 ± 0.9	9.3 ± 2.1 *	7.3 ± 0.8	7.3 ± 1.3
	Females	6.9 ± 1.6	8.1 ± 2.3	6.8 ± 0.6	6.5 ± 1.2	7.3 ± 1.0	10.6± 0.8 *^,€,#,‡^	6.6 ± 0.7	6.4 ± 1.0
Glucose AUC	Males	2772 ± 492	2785 ± 446	2490 ± 377	2608 ± 309	2308 ± 231	2519 ± 512	2048 ± 325	1917 ± 219
	Females	1756 ± 361	2079 ± 553	1666 ± 391	1851 ± 546	1648 ± 267	2270 ± 96 *	1387 ± 190	1882 ± 349 *

Note: Values are presented as means ± SD. * significantly different over time; ^€^ significantly different from CON; ^#^ significantly different from HIIT; ^‡^ significantly different from IF + HIIT.

**Table 2:** The effect of 12-weeks of intermittent fasting and high intensity exercise combined or individual on plasma TAG, LDL and HDL in male and female mice.

Variables	Males					Females				
	OBC	CON	HIIT	IF	IF + HIIT	OBC	CON	HIIT	IF	IF + HIIT
TAG (mg/dL)	60.1 ± 14.6	58.9 ± 10.7	72.1 ± 16.5	62.7 ± 10.6	57.0 ± 27.7	65.9 ± 20.5	70.9 ± 13.1	75.6 ± 14.8 ^‡^	66.5 ± 15.7	51.3 ± 24.7 ^#^
HDL (mg/dL)	69.8 ± 12.2	76.2 ± 11.6	68.7 ± 11.3	82.1 ± 32.1	72.1 ± 17.3	51.1 ± 9.0	65.1 ± 23.0	61.1 ± 9.5 *	58.6 ± 9.1 *	50.8 ± 7.7
LDL (mg/dL)	40.5 ± 17.7	82.3 ± 36.0 *^,‡,§^	74.8 ± 15.6 *^,‡,§^	40.4 ± 16.9 ^€,#^	29.9 ± 8.9 *^,€,#^	40.5 ± 17.7	24.8 ± 11.8 *	17.8 ± 4.8 *	19.5 ± 6.0 *	27.0 ± 8.9 *

Note: Values are presented as means ± SD. * significantly different over time; ^€^ significantly different from CON; ^#^ significantly different from HIIT; ^§^ significantly different from IF; ^‡^ significantly different from IF + HIIT, OBC-obese baseline control measured at the end of 12-week HF/S period (0-week).

**Table 3:** The effect of 10-weeks of intermittent fasting and high intensity exercise combined or individual on absolute and relative muscle strength in both male and female mice.

Variables	Gender	CON		HIIT		IF		IF + HIIT	
		0 Week	10 Week	0 Week	10 Week	0 Week	10 Week	0 Week	10 Week
Absolute muscle strength (Score) ^#^	Males	9.4 ± 1.8	10.5 ± 1.7 *	10.1 ± 1.2	10.6 ± 1.4	10.7 ± 1.6	11.2 ± 0.9	10.5 ± 1.1	11.1 ± 1.3
	Females	9.7 ± 2.7	9.8 ± 2.6	10.8 ± 1.9	11.3 ± 2.3	12.1 ± 1.9	9.4 ± 1.6 *	11.8 ± 1.9	12.2 ± 1.6
Relative muscle strength (Score/LM) ^$^	Males	0.33 ± 0.05	0.35 ± 0.05	0.37 ± 0.04	0.38 ± 0.05	0.37 ± 0.05	0.39 ± 0.04	0.37 ± 0.05	0.39 ± 0.05
	Females	0.44 ± 0.12	0.42 ± 0.10	0.50 ± 0.10	0.50 ± 0.11	0.52 ± 0.11	0.40 ± 0.09 *	0.54 ± 0.09	0.53 ± 0.08

Note: Values are presented as means ± SD. ^#^ Absolute muscle strength score represents maximum muscle strength produced independent of muscle or body size; ^$^ Relative muscle strength represents absolute muscle strength divided by lean mass (LM); * indicates significant changes over time.
